# Implicit and Explicit Consumer Perceptions of Cashews: A Neuroscientific and Sensory Analysis Approach

**DOI:** 10.3390/foods14071213

**Published:** 2025-03-30

**Authors:** Rocio Lopez-Navarro, Luis Montero-Vicente, Carmen Escriba-Perez, Juan M. Buitrago-Vera

**Affiliations:** Department of Economics and Social Science, Universitat Politècnica de València, Camino de Vera w/o No., 46022 Valencia, Spain; luimonvi@upv.es (L.M.-V.); carespe@upv.es (C.E.-P.); jmbuitrago@upv.es (J.M.B.-V.)

**Keywords:** cashews, consumer neuroscience, sensory analysis, consumer food preferences, implicit measures, explicit measures, EsSense Profile^®^

## Abstract

This study investigated consumer perceptions of raw cashew nuts from two different private labels (private label A, PLA, and private label B, PLB), employing a combination of explicit (sensory analysis) and implicit (consumer neuroscience) methods. The objective was to analyse both conscious and unconscious responses to understand consumer preferences. Participants (n = 80) evaluated the samples, with electroencephalography (EEG) and electrodermal activity (EDA) as implicit methods, and hedonic scales, JAR scales, and the EsSense25 questionnaire used for explicit evaluations. The results revealed a clear preference for PLB, supported by higher global hedonic scores and a significant majority (65%) choosing PLB over PLA. EEG metrics calculated for participants’ valence, frontal alpha asymmetry (FAA) for flavour indicated greater activity in the left frontal lobe for PLB, associated with positive emotions. Task engagement (TE) measurements revealed higher engagement with PLB during flavour evaluation. Penalty analysis identified that PLA was mainly penalised for a “too weak” aroma and flavour. The EsSense25 analysis showed that cashew consumption evoked predominantly positive emotions such as “pleasant”, “satisfied”, and “calm”. In conclusion, the combination of implicit and explicit methods provided a comprehensive understanding of consumer preferences, highlighting the value of both approaches and the importance of sensory attributes in driving the overall liking of raw cashews. The findings have implications for product optimisation, market segmentation, and the development of marketing strategies in the cashew industry.

## 1. Introduction

Cashew nuts have gained increasing economic, nutritional, and consumer importance in recent years [1,2]. From an economic perspective, the global cashew nut market was valued at USD 5.95 billion in 2023, with a projected annual growth of 5.4% until 2030 [3,4]. This growth is largely due to increasing awareness of their nutritional benefits. They are also a rich source of monounsaturated fats, protein, vitamins and essential minerals such as magnesium, phosphorus, and zinc, which contribute to brain and cardiovascular health [5,6]. In addition, recent studies have shown that regular consumption of cashew nuts can help lower LDL cholesterol and triglycerides, thereby reducing the risk of cardiovascular disease [7]. However, as with many nuts, cashews have been found to contain mycotoxins, which may present a chemical hazard and compromise human health [8]. Studies have indicated that the concentrations of these toxins are generally lower in cashews when compared to other nuts, such as peanuts or walnuts, and that they do not pose a significant food safety risk [9,10]. Nevertheless, this parameter must be considered when evaluating the quality of this product in order to ensure that mycotoxin levels are safe for consumers. From a consumer perspective, the perception of cashews as a healthy and versatile snack has driven their demand, especially among health-conscious consumers seeking nutritious alternatives to traditional snacks [11,12]. This combination of economic, nutritional, and consumer preference factors has positioned cashews as a food of growing importance in the global health food market [13,14].

The development of novel foods depends, to a large extent, on knowing consumer preferences, which are influenced by a complex set of emotions, attitudes, or values that are difficult to measure. Some of the existing methods, such as questionnaires or interviews, generally measure conscious and rational responses to any stimulus by relying on different tools, such as the use of response scales, such as hedonic or just-about-right scales, when the objective is to assess consumer preferences [15,16]. But these methods have limitations, as they depend on people’s ability and willingness to accurately report their attitudes and/or motivations, leading to biases in many cases, especially when the responses expected to be collected have a high emotional component [17]. Therefore, unconscious measures are increasingly used to assess consumer response, as they provide a more comprehensive view of people’s reactions. The physiological mechanisms that consumers present in response to a stimulus can be so fast that they themselves are not aware of them; thus, through different neuroscience techniques, it is possible to measure emotions and spontaneous reactions of consumers and reflect them in a more unbiased and meaningful way [18].

Consumer neuroscience is a discipline that studies behaviour in response to a stimulus using biometric techniques [19], such as electrodermal activity (EDA), electroencephalography (EEG), eye tracking, and heart rate measurement. These tools can provide simple psychophysiological measures, such as EDA, which simply measures changes in skin conductance produced by the excitation of the sympathetic nervous system and directly associated with variations in the electricity transmitted by nerves and sweating [20]. An increase in skin electrical conductance signals is associated with increased arousal, generated by a state of alertness in the body, and is therefore used as an indication that the stimulus provokes an emotion in the consumer. Conversely, a decrease in this parameter reflects a lack of arousal [21].

EEG is a non-invasive technique that measures electrical changes in the cerebral cortex, providing information about emotional states [22]. This technique is distinguished by having a high temporal resolution in milliseconds [23] that accurately measures the response to changing stimuli. EEG measures the subject’s brain activity to subsequently determine frontal alpha asymmetry (hereafter FAA) as a marker of affinity or rejection of stimuli (valence) [24]. FAA measures changes in the alpha brain rhythm (8–13 Hz) of the frontal lobe and is based on the fact that emotions related to rejection are detected in the right hemisphere while emotions associated with acceptance are detected in the left hemisphere [25]. These unconscious methods must be combined with conscious methodologies, such as questionnaires or other commercial research techniques [26].

On the other hand, to identify the motivations or emotions evoked by food, there are psychographic methods that measure consumers’ emotional responses, such as the EsSense Profile^®^. The EsSense Profile^®^ was specifically designed for the emotional evaluation of food. It consists of a questionnaire developed by [27], which includes 39 emotions classified as positive, negative, or neutral. After consuming the food, consumers are asked to identify which emotions they experienced by marking their responses on the questionnaire.

In recent years, the integration of explicit methodologies—such as questionnaires and the EsSense Profile—with implicit methodologies for studying food consumers has resulted in numerous studies across a wide variety of food products. For example, [28] compared three cider brands, two of which had a protected designation of origin and one that did not, using eye-tracking data analysis (EDA) and the EsSense Profile questionnaire. Similarly, [29] investigated consumer responses to five beer samples in different contexts, including a laboratory and a restaurant setting.

A study [30] focused on beverages, specifically various types of vegetable juices, and examined how purchase intention affects consumers’ choices. Research on solid foods has examined chocolate pudding, focusing on consumer reactions to varying sucrose concentrations [31], as well as the effects of presentation formats for textured foods targeted at the senior population [32]. Additionally, some studies have measured implicit responses during different cooking phases across various meals [33,34] and explored consumer reactions to unfamiliar meals [35].

Despite the complexity and cost associated with neuroscientific techniques such as EEG (electroencephalography) and EDA (electrodermal activity), their application in consumer research is becoming increasingly justified. As technology advances, these tools are becoming more affordable and user-friendly, making them more accessible to both researchers and marketers [26].

Furthermore, the use of these techniques aligns with a growing trend in consumer food research that seeks to move beyond simple product liking and to explore a deeper understanding of consumer preferences [15]. By providing objective measures of unconscious responses, EEG and EDA offer valuable and reliable insights that complement traditional explicit measures [36]. This allows for a more comprehensive understanding of consumer behaviour.

This holistic approach is particularly relevant in the competitive food market, where recognising subtle differences in consumer perceptions can provide a significant advantage.

As mentioned previously, using both explicit and implicit methodologies in food consumer research is increasingly common; however, there is a lack of studies focusing specifically on nuts, especially cashews. This gap is significant given the increasing importance of nuts in the global health food market. Our study addresses this void by examining consumer perceptions of raw cashews from two supermarket private labels, using a combination of neuroscience techniques (EEG and EDA) and traditional explicit methods.

This approach contributes to the broader field of food consumer research and offers valuable insights that were previously unavailable, specifically for the nut industry. By applying these advanced techniques to cashew research, we create new opportunities to understand consumer preferences within the often-overlooked nut sector, which could lead to more effective marketing strategies.

Finally, the aims of this study were as follows:

- Examine the consumer’s unconscious response with two neuroscience techniques, EEG and EDA;
- analyse the consumer’s conscious response, both emotionally and in terms of acceptance;
- distinguish whether there are differences in consumer perception, both conscious and unconscious, between two private labels from to different supermarkets;
- analyse the possible food marketing implications of the combined use of implicit neuroscience-based methods in conjunction with explicit methods.

## 2. Materials and Methods

### 2.1. Participants

Following approval by the Institutional Review Board of Universitat Politècnica de València (P11_26-09-2023) in València, a total of 80 participants (18–64 years old and 54% female) including students and staff were recruited by means of convenience sampling. Because the study used techniques that collect neurophysiological data, participants should not be under treatment with any psychotropic drugs, should not suffer from neurological diseases [33], and should be right-handed to obtain a more homogeneous sample, because hemispheric specialisation in the cerebral cortex is different in left-handed individuals [37]. On the other hand, they also had to be non-smokers and not allergic to any type of nut.

All participants signed an informed consent and personal data protection before attending the tasting session, as required by the Declaration of Helsinki of the World Medical Association (WMA, 2013 [38]) and EU Regulation 2016/679 of the European Parliament and of the Council of 27 April 2016 on the protection of individuals with regard to the processing of personal data and the free movement of data and Organic Law 3/2018 of 5 December on the Protection of Personal Data and guarantee of digital rights. Finally, participants received a EUR 15 gift card for a Spanish department store as a reward for their collaboration in the study.

### 2.2. Sample Preparation

For the study, raw cashew nuts of two private label brands (PLA and PLB) with similar quality were purchased weekly from two different well-known local supermarkets. Once the cashew packs were opened, they were not used for more than 5 days, as recommended by the manufacturer on their packaging. As this was a blind test, all samples were served in white 60 mL cups coded with three random digits so that consumers could not identify them [39].

### 2.3. Implicit Methodologies: Applied Neuroscience Techniques

The techniques used in this study were electroencephalography (EEG) and electrodermal skin activity (EDA), both of which provided us with the physiological and unconscious responses of the participants. The responses of both were monitored and recorded using iMotions software version 9.3 (iMotions A/S, Copenhagen, Denmark).

EEG signals were measured using an Enobio 8 electroencephalography device (Neuroelectrics, Barcelona, Spain), which recorded brain electrical waves at a sampling frequency of 500 Hz for subsequent processing and calculation of the metrics detailed in the following section. The EEG device was used with a 7-channel frontal configuration at points determined by the international 10/20 system in the orbitofrontal (F7/F8), prefrontal (AF7/AF8), frontopolar (Fp1/Fp2), and frontal midline (Fz) areas, arranged in a neoprene band, so that the adjustment to the participant’s head was adequate.

The electrodermal skin activity study, hereafter EDA, is a technique that measures changes in skin conductance (µS) produced by the involuntary response of the autonomic nervous system to a stimulus [40]. A SHIMMER™ device (SHIMMER Research Ltd., Dublin, Ireland) was employed at a sampling rate of 128 Hz, which consisted of a controller placed on the wrist of the non-dominant hand, from which two wires were connected to two 40 mm diameter AgCl pregelled electrodes and placed on the palm of the hand, specifically on the thenar and hypothenar eminences, areas where a greater number of sweat glands are found [41,42,43].

### 2.4. Explicit Methodologies: Affective Sensory Testing

In order to collect data from the explicit methodologies, a questionnaire was designed for participants to fill in parallel to the EEG and EDA data collection while evaluating the samples, including affective tests of acceptance and preference, as well as other emotional and socio-demographic aspects.

The structure of this consisted of five sections: the first three to assess visual appearance, aroma, and flavour, followed by overall liking and choice between the two private labels, and, finally, the EsSense25, a reduced version of EsSense Profile^®^ [44].

The first section included questions related to visual attributes of both private labels. Acceptance of the appearance of each private label was assessed on a 9-point hedonic scale (1 “I don’t like it at all”, 9 “I like it very much”) and, using 5-point verbal JAR scales, on size (1 “Too small” …, 5 “Too big”).

The second section asked about the hedonic acceptability of the aroma of each of the samples and the intensity of the aroma using a JAR scale (1 “Too weak”, 5 “Too intense”). The third section consisted of a question on overall flavour acceptability, with the same 9-point hedonic scale used for appearance and aroma and two further questions with a JAR scale for flavour intensity (1 “Too weak”, “5 “Too intense”) and texture (1 “Too soft”, 5 “Too hard”).

Finally, the overall liking of both samples was assessed using a 9-point hedonic scale as outlined above, followed by a bilateral paired comparison test, according to the standard ISO 5495 [45], wherein participants had to choose which of the two private labels they preferred after having tried them. Finally, the emotional responses were assessed using the EsSense25 framework with a check-all-that-apply (hereafter CATA) question included with the twenty-five EsSense25 emotions. The age range and gender of the participants were also recorded.

### 2.5. Consumer Session Procedure

The experiment was conducted within the facilities of the Universitat Politècnica de València, in a room set up for this purpose, with the appropriate temperature and lighting and free of external odours and noise [39]. This space was close to another sample preparation room but sufficiently far away to avoid interference with the study participants.

Once the consumers who met the requirements for an individual session lasting about 45 min had been summoned, they were sent an information sheet with the procedure and the neuroscience devices that were going to be used. Thus, once the participant arrived at the facility, after a brief explanation of the procedure and resolution of any doubts, the informed consent form was provided for signature. The participant was then seated in a chair in front of a screen that would show instructions to be followed at all times. Afterwards, the EEG and EDA devices were placed, and the signal from both was checked to ensure that it was correct to begin the study.

**Figure 1:**
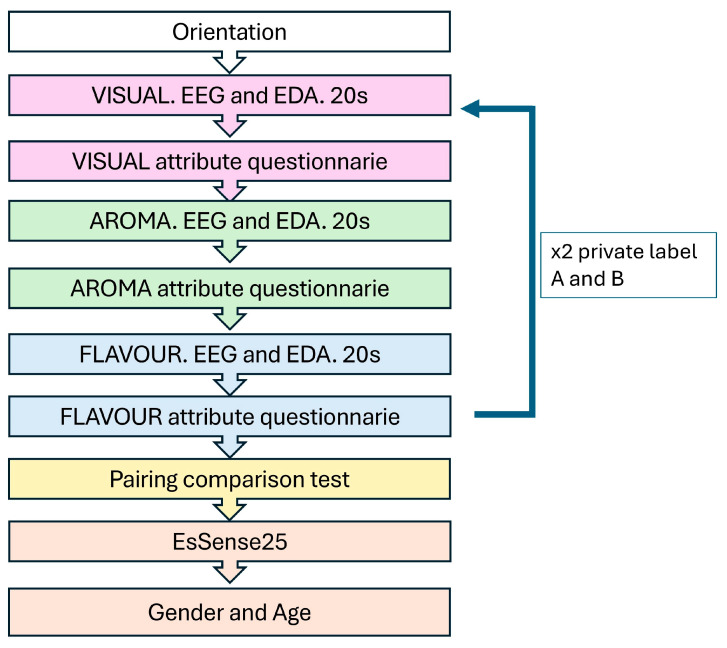
Graphical representation of the procedure followed in each session with the 80 consumers participating in the study.

The experiment consisted of 5 distinct phases (Figure 1), which are described below.

The first three phases of the experiment corresponded to studying the perception of the two private labels described above (PLA and PLB) with neuroscience techniques. In these phases, the samples were presented in a sequential monadic manner, randomising the order of sample presentation per participant [39]. Finally, there was a fifth phase where participants’ self-reported emotions were collected using the EsSense25 instrument.

First, the “Visual Phase” began with participants observing the external appearance of each sample for 20 s and then proceeding to evaluate them using the hedonic and JAR-type scales described in Section 2.4. The next phase consisted of assessing the aroma of the samples (“Aroma Phase”). In this phase, participants had to smell each sample for 20 s and then answer the aroma questions. The third phase was the evaluation of the flavour of the samples. Although it was similar to the previous ones, it had to be adapted to be able to capture the EEG signals adequately, as chewing interfered with the signal, and it was not possible to eliminate it during processing. This adaptation consisted of instructing the participant to taste the sample by chewing normally and then to remain at rest for 20 s to ensure that the EEG measurements were correct. As in the previous two phases, the flavour of both samples was assessed using hedonic scales and JAR.

The fourth phase consisted of the participants’ overall liking of the two samples using a 9-point hedonic scale. Once they had rated the samples, they were asked to select their preferred sample, following the paired comparison test procedure described in Section 2.4.

Subsequent to the collection of the EEG and EDA signals, along with the sensory attributes for both samples obtained from the questionnaire, the fifth and final phase was initiated. This consisted of consumers answering the last question of the questionnaire, which corresponded to the EsSense25. For this, participants were asked to select the emotions they most identified with after consuming the raw cashew nuts through a check-all-that-apply (CATA) question. This question allowed consumers to choose from a list of twenty-five emotions, thereby providing insight into their emotional experiences associated with the consumption of the cashew nuts. Finally, participants were asked about their age and gender.

### 2.6. Data Processing

#### 2.6.1. Electroencephalography (EEG)

The EEG data were processed according to the methods described by [46]. First, frequencies between 0.5 and 100 Hz were filtered using a Butterworth bandpass filter with zero phase delay, followed by a 50 Hz notch filter to mains noise. All values above 120 uV were then removed from the electrical waveform, as the typical EEG signal of an adult human being is between 10 and 100 uV. Once the signal was processed, the power spectral density (PSD) was calculated using fast Fourier transform (FFT) at theta (4–8 Hz) and alpha (8–12 Hz) frequencies for the calculation of the following metrics:

- Frontal alpha asymmetry (FAA): This was used to compare consumer valence towards the two private label of raw cashew nuts. It is the result of the difference of the Napierian logarithm of the power spectral density between the right and left frontal lobe [24], according to the following equation:

$$\mathrm{FAA}=\ln\alpha PSD\left(F8,AF8,Fp1\right)-\ln\alpha PSD\;\left(F7,\;AF7,Fp1\right)\;$$

This metric is based on the theory that higher activity in the right frontal lobe is associated with negative emotions, while higher activity in the left frontal lobe is associated with positive emotions [47]. In the case of the alpha rhythm (8–13 Hz), an increase in alpha rhythm is inversely related to frontal lobe activity, i.e., a decrease in alpha frequency potential in the right frontal lobe implies greater activation in the left frontal lobe, and vice versa [48]. Higher FAA values indicate greater activation of the left frontal lobe, which is associated with approach motivation and a stronger affinity for the stimulus. Conversely, lower FAA values suggest greater activation of the right frontal lobe, indicating avoidance motivation and lower affinity for the stimulus. It is essential to interpret FAA scores in a relative context, comparing them across different stimuli to determine which one elicits stronger approach or weaker avoidance motivations [24,33,48]. FAA is a dimensionless metric.

- Task engagement (TE): Measures the participant’s cognitive state in response to a stimulus, indicating their level of involvement or mental effort regarding this stimulus [49,50]. For this purpose, frontal midline activity (Fpz) will be monitored at the theta frequency (4–8 Hz), calculating its power spectral density in dB using the procedure described at the beginning of this section. An increase in theta power is directly related to an increase in cognitive workload, which results in greater interest, attention, or mental effort towards the task being performed at that moment [51].

#### 2.6.2. Electrodermal Skin Activity, EDA

The conductance signals (µS) obtained for the study of the electrodermal activity were analysed using continuous decomposition analysis (hereinafter CDA). CDA consists of decomposing the signal of the skin conductance data into continuous signals of phasic (activation) and tonic (basal state) activity. This method takes advantage of the recovery of the signal characteristics of the underlying sudomotor nerve activity [40]. Prior to this analysis, the signal was pre-processed by applying a low-pass Butterworth filter (cutoff frequency: 5 Hz) to remove high-voltage line noise. Subsequently, the CDA considered all signals with an amplitude above 0.0005 µS and a duration above 500 ms as stimulus responses.

The metric calculated for each stimulus was the number of skin conductance response peaks per minute (nSCR), which is described as the number of detected peaks (phasic signal, dimensionless) that the consumer had during this stimulus [40] and has been widely used by different authors to study the intensity of the emotion elicited in the consumer [20,28,33].

#### 2.6.3. Statistical Analysis

Statistical analyses of the obtained data are described below. All statistical analyses were conducted using the XLSTAT Premium, version 2024.4.0, statistical package (Addinsoft, Paris, France).

For the metrics obtained using EEG (FAA and TE) and EDA (nSCR) and the ratings obtained using hedonic scales, the Wilcoxon signed-rank test, a non-parametric test indicated for two related samples [52], was used, as the distribution of the data was non-normal. The Shapiro–Wilk test was used to check the normality of the data and showed that they were not normal. For both tests, the significance level was 5% (*p* < 0.05).

On the other hand, for the significance of the bilateral paired comparison test, where consumers had to choose which of the two samples they preferred overall, the tables in Appendix A of ISO 5495 [45] were used. In the case of this study, where the test was bilateral, when α = 0.05 and n = 80, at least 50 consumers (62.5%) should select the same sample as their preferred sample.

The JAR data collected were analysed in two phases for each nut: a frequency analysis and then a penalty analysis.

For the frequency analysis, the frequencies marked as low (1 “Too much little” and 2 “Too little”) were grouped for each attribute analysed and expressed as a percentage (%). The same was performed for the ideal point, JAR (3 “Just About Right”), and for the high level (4 “Too much” and 5 “Much Too much”) to obtain a bar chart showing the percentages of the three levels obtained, which were named “Too little”, “JAR”, and “Too much”.

Once the relative frequencies (in %) had been obtained, the next stage in dealing with the JAR data was the penalty analysis. This analysis relates the JAR data to overall product liking and aims to measure whether attributes that consumers have found to be high or below their ideal point produce a significant penalty in overall liking. To arrive at this, the overall liking scores for each level mentioned above (“Too little”, “JAR”, “Too much”) were grouped and averaged. Penalties or mean drops were then obtained for the “Too much” and “Too little” levels by subtracting them from the average of the “JAR” level.

Once the mean drops were obtained, they were standardised, and a 3-level mean comparison test was performed at a 5% significance level to detect which penalties were significant and influenced the overall liking of the product.

Subsequently, to gain a better understanding of the data obtained in this analysis, a penalty graph was created. In this graph, the penalties calculated for each attribute are plotted against the percentage of consumers at each level.

Finally, the data collected using the EsSense25 tool were processed qualitatively, creating a ranking with the frequency (in %) with which each emotion was marked in order from highest to lowest. In order to make it less complex to see which emotions stood out the most, they were represented by a radial graph, thus generating the emotional profile of each one.

It is important to acknowledge the limitations of this study. The focus on only two private label brands within the same product category restricts the analysis of biometric measures to paired comparison tests for related samples [53]. To address this limitation and provide a more comprehensive understanding of consumer preferences, we have expanded our analysis to include additional approaches involving explicit measures, such as penalty analysis.

## 3. Results

### 3.1. Implicit Measures: Unconscious Consumer Response

Table 1 and Table 2 show the average of the metrics calculated from the EEG and EDA signals recorded from the 80 consumers. Metrics were calculated to measure valence, arousal, and attention for the appearance, aroma, and flavour of each sample.

Regarding the measures related to valence (FAA) and arousal (nSCR) (Table 1), after performing the Wilcoxon test for related samples, significant differences were found, mainly in aroma and flavour. Appearance showed no differences in any of the calculated metrics (*p* = 0.869 and *p* = 0.519, respectively).

Specifically, higher arousal (*p* < 0.0001), by nSCR measurement, was observed for PLB, so it could be deduced that consumers showed greater emotional intensity for the aroma (nSCR = 8.139) of this private label. The valence data measured with FAA were not significant (*p* = 0.106).

Regarding flavour, consumers’ preference was also for PLB, as it has a higher FAA value (−0.241), which implies a higher affinity for it. The metric calculated for skin conductance (nSCR) showed no significant differences between private labels, so the arousal level was similar among consumers.

In addition to valence and arousal, task engagement (TE) was also calculated using EEG by monitoring theta frequency activity (4–8 Hz) in each phase of the test and for each private label (Table 2). In this case, there were significant differences for aroma (*p* < 0.001) and flavour (*p* = 0.037). For aroma, a slight increase in involvement was observed in PLA. In contrast, for flavour, higher involvement is seen with PLB. These results reflect that in both the aroma and flavour of both private labels, there are attributes that have captured the attention of consumers, although the motivations leading to this are unknown.

To complement what has been obtained at the implicit level, as well as to specify which possible attributes are behind consumers’ unconscious choices, the following section describes the results of the explicit methodologies based on sensory analysis that have been used in this research.

### 3.2. Explicit Measures: Conscious Response

#### 3.2.1. Hedonic Scales and Paired Comparison Test

In addition to measuring their unconscious response, consumers were asked to rate the two samples to be studied (PLA and PLB) on a nine-point hedonic scale on the following attributes: appearance, aroma, flavour, texture, and overall liking; the results were then compared using the Wilcoxon non-parametric signed-rank test (Table 3).

Of these, only aroma (*p* = 0.001) and overall liking (*p* = 0.017) showed significant differences, and both showed higher values for PLB (Table 3). Appearance (*p* = 0.120), flavour (*p* = 0.060), and texture (*p* = 0.065) showed no significant differences.

To determine which private label of raw cashew nuts they preferred, consumers had to choose between the two samples presented following the procedure of a bilateral paired comparison test, as described in the ISO 5495 standard [45]. Table 4 shows the number of consumers who chose each sample. Of the 80 consumers who participated in the study, 52 (65%) chose PLB compared to 28 (35%) consumers who chose PLA. According to Table A2 of Appendix A of ISO 5495 [45] and for an alpha = 0.05, these results were statistically significant, which means that there is a clear preference for the PLB cashew sample.

Considering these results, aroma and flavour were the attributes that determined the preference for PLB. In order to identify to what extent these attributes have influenced the overall liking, the following section describes the results obtained by means of the penalty analysis.

#### 3.2.2. Penalty Analysis

JAR scales were used to evaluate five attributes of raw cashew nuts (size, colour, aroma, flavour, and texture). This was performed to see which attributes penalise the overall liking of the product the most, as well as to further investigate consumer opinion and find possible points of improvement for the product.

**Figure 2:**
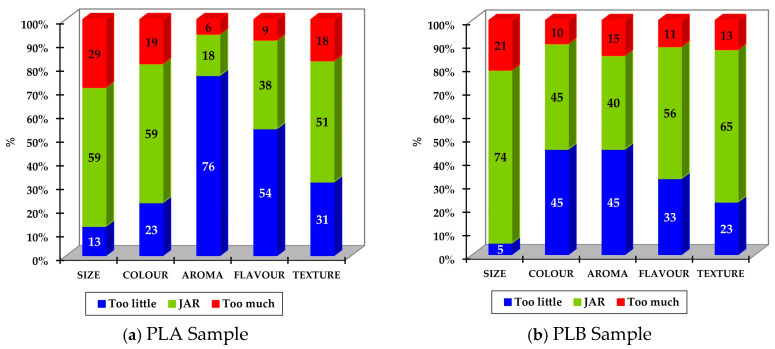
Percentage of consumers aggregated according to three levels of intensity (too little, JAR, too much) for each attribute and for each private label of raw cashew nuts.

Figure 2a,b corresponds to the results grouped into three levels (too little, JAR, and too much) for each sample and the attributes analysed, using the procedure described in the methodology; the difference between the perception of aroma and flavour between the two private labels stands out. In the case of the PLA sample, 76% (Figure 2a) of consumers perceived the aroma of raw cashew nuts to be too weak for their liking, compared to 40% for PLB (Figure 2b). Regarding flavour, 54% of consumers found it less intense than they would like in the case of PLA, while for PLB, it was 36%.

**Figure 3:**
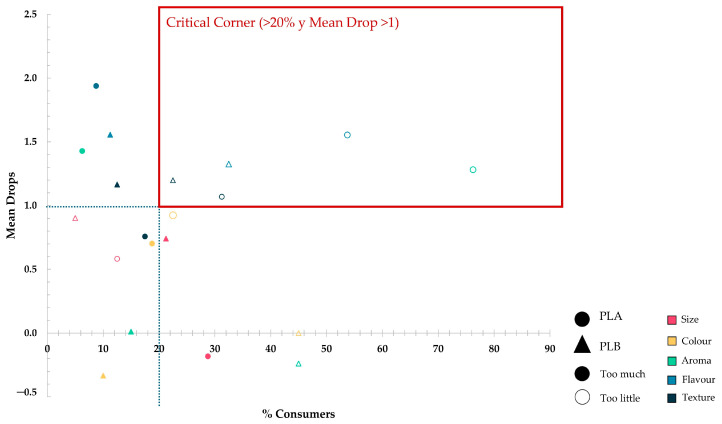
Graphical representation of the penalty analysis conducted for both private labels of raw cashew nuts. The dotted line delimits the mean drops greater than one on the *x*-axis and the percentage of consumers who have marked each level greater than 20% on the *y*-axis to obtain the critical corner (red lines).

Once an initial frequency analysis was performed, the results of the penalty analysis were obtained, where the calculated mean drops were assessed for significance using multiple pairwise comparisons, as described in the methodology. The tables with the significance tests for each sample and attribute measured can be found in Appendix A. To better understand these results, a penalty graph was also created (Figure 3), which is the result of plotting the mean drops for the “Too little” and “Too much” levels against the percentage of consumers who had marked each one.

To visually detect which attributes might require attention to improve both private labels of cashew nuts, the “critical corner” was delimited in the graph of penalties, which includes the attributes with mean drops higher than 1 and whose frequency exceeded 20% of consumers. Figure 3 shows that for PLA, the critical attributes are aroma (*p* = 0.002), flavour (*p* < 0.0001), and texture (*p* = 0.002), which are perceived as too weak in all three cases. For the PLB, the following penalised the overall liking: flavour (*p* < 0.0001) and texture (*p* = 0.002), perceived as too weak and too soft, respectively.

#### 3.2.3. Emotional Profile of Raw Cashew Nuts. EsSense25

**Figure 4:**
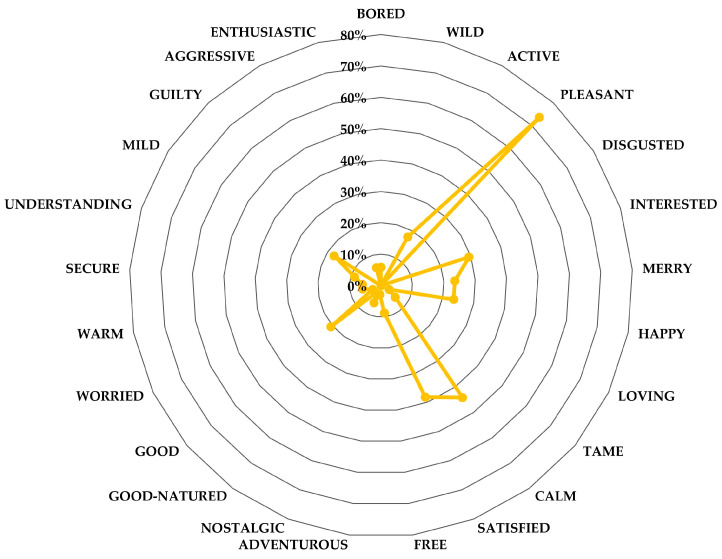
Radial representation of the emotional profile of raw cashew nuts using the EsSense25 lexicon.

For the analysis of the EsSense25 lexicon, only emotions marked with a frequency of more than 20% were considered [24,30]. Figure 4 shows the frequency of emotions they identified with after consuming raw cashew nuts. It can be seen that positive emotions such as “Pleasant” (71.25%), “Satisfied” (50%), “Calm” (45%), or “Happy” (25%) predominate, which is common for emotions associated with food.

On the other hand, EsSense25 has also been useful when detecting participants with anomalous behaviour, such as those who had bad experiences with cashew nuts or other nuts, which could evoke negative feelings and influence the overall analysis. This was not the case in this study, as the number of negative emotions was negligible, less than 3%.

## 4. Discussion

### 4.1. Comparison of Explicit vs. Implicit Methods

This research aimed to analyse consumers’ conscious and unconscious responses to raw cashew nuts from two private labels (PLA and PLB). Implicit methods based on consumer neuroscience techniques and explicit methods such as sensory analysis were used.

The results reveal a clear preference for the PLB sample. The overall preference for PLB is evident in both implicit objective measurements using neuroscience techniques and subjective evaluations. Overall hedonic scores were significantly higher for PLB (Table 3), and the preference test revealed that a significant majority of consumers (65%) chose PLB over PLA (Table 4). These findings align with previous studies highlighting the importance of sensory as emotional experience in consumers’ food acceptance and choice [30,54,55].

At the implicit level, the higher approach for PLB was reflected in the FAA values for flavour, indicating greater activity in the left frontal lobe, associated with positive emotions. This result suggests that the sensory experience with PLB generated a more favourable emotional response in participants, even at an unconscious level. Although no specific literature studying consumer perception of nuts using EEG was found, other studies have employed FAA as an indicator to examine valence for certain types of foods or related experiences. For instance, [37] found significant differences when comparing videos of different breakfast meals under varying hygienic conditions, specifically observing greater activity in the right frontal lobe for those presenting deterioration compared to the control, implying that consumers responded negatively to this stimulus as expected. Another study [33] reported similar results when comparing the FAA of consumers while cooking chicken and mealworms, with the latter resulting in lower FAA values, thus indicating greater rejection. The same authors repeated a study using two stimuli deemed pleasant [34], where consumers prepared the same Thai chicken meal with different quality ingredients—premium and basic. In this instance, none of the EEG implicit parameter measures employed were significant. This study is similar to ours in that the samples were comparable, and consumers were not shown the product brand. However, unlike their findings, we discovered significant differences in flavour (Table 1). This suggests that FAA could be useful for comparing two similar stimuli, a finding that warrants further verification through additional studies involving different types of nuts or other foods.

Task engagement (TE) is a metric based on measuring the brain activity of theta waves (4–8 Hz), frequencies whose increase in the frontal lobe is related to processes where cognitive effort is higher and our brain is allocating more resources [56]. In this study, significant differences in participants’ attention and motivation during aroma and flavour evaluation were revealed (Table 2). A slight increase in engagement with PLA was observed during aroma evaluation, which might indicate that this attribute generated a higher cognitive processing need in the participants. However, the higher involvement with PLB during flavour evaluation suggests that this attribute was more motivational for consumers. Other authors such as [35] conducted research on food neophobia, where participants with higher food neophobia also showed higher levels of attention for an unfamiliar food product, aligning with the relationship between theta wave activity and mental workload. In our study, both stimuli were familiar to consumers. As noted earlier, the main differences lie in factors related to the aroma and flavour of both samples, which require greater attention, regardless of their valence.

Regarding the EDA metric, nSCR, significant differences were observed only in aroma, which provides limited insight into the arousal levels in both samples. Other studies, such as [33,34], have reported similar findings while studying familiar samples among consumers, where they also utilised EDA to examine arousal. In the first case study (mealworms vs. chicken), there was greater arousal in some phases of mealworm cooking, but in the second study where the samples were similar, no significant differences were found. Other authors found the same when studying responses to mixed vegetable juices [30], as did [57] with different types of beers.

On the other hand, when marketing variables such as brand or quality are included in experiments, more differences in consumer arousal exist. In a study conducted by [58] that examined private labels, specifically in the context of chocolate, researchers compared these private labels with national brands. They found that differences in arousal occurred only when consumers were aware of the brand. Under blind conditions, however, consumers demonstrated the same emotional response regardless of the brand. Also, brand familiarity played a significant role in the study conducted by [28], which examined how consumers perceive various types of cider with a protected designation of origin (PDO). The study found that consumers had a stronger emotional response when they were unfamiliar with the PDO logo compared to when they recognised it. These findings suggest that techniques like EDA may yield more insightful results when consumers have more information about a product. Therefore, it would be interesting for future studies on nuts to explore how knowing specific attributes—such as the brand or nuts origin—affects emotional responses in consumers.

Penalty analysis (Figure 3) revealed that the main deficiency of PLA was the perception of an aroma and flavour that was “too weak”. In contrast, PLB was penalised for its flavour and texture being “too weak/soft”, although to a lesser extent. This suggests that PLB achieved a more balanced and satisfying sensory profile for consumers, although there are still opportunities for improvement in flavour intensity and texture firmness.

The EsSense25 analysis revealed that raw cashew nut consumption predominantly evokes positive emotions, such as “Pleasant”, “Satisfied”, “Calm”, and “Joyful” (Figure 4). This positive emotional profile aligns with the general perception of nuts as healthy and enjoyable snacks [11,59]. The absence of significant negative emotions suggests that the sensory experience with cashew nuts was generally well received by participants.

Consequently, aroma and flavour stood out as the key sensory attributes that differentiated the two private labels and guided the preference for PLB. Results from the hedonic scales showed significantly higher scores for aroma in PLB (Table 3), consistent with higher arousal as measured by skin conductance (nSCR) (Table 1). This finding underlines the importance of aroma as a determining factor in food perception and acceptance [60,61,62].

### 4.2. Marketing Implications

The findings of this research have important marketing implications for the raw cashew nut industry as well as for the development of novel foods:

1. Optimising the sensory experience: Raw cashew nut manufacturers can use the results of this study to optimise the sensory profile of their products, paying particular attention to aroma and flavour, to maximise consumer acceptance and preference. The information provided by the penalty analysis can guide the formulation of products with an intensity of aroma, flavour, and texture that matches consumer expectations and preferences.
2. Market segmentation: The information obtained in this study can be used to segment the raw cashew nut market based on consumers’ sensory and emotional preferences. This would allow companies to tailor their products and marketing strategies to the specific needs and desires of each market segment.
3. Marketing strategies: Companies can use the findings of this research to design more effective marketing and communication strategies that highlight the sensory and emotional attributes of their raw cashew nut products. For example, advertising messages could be created that emphasise the aroma and flavour of cashew nuts or associate the consumption of cashew nuts with positive emotions.
4. Novel food development: Understanding the underlying emotions and preferences of consumers, gained through neuroscience techniques, can be used for the development of new food products that meet consumers’ needs and desires more effectively. For example, products could be developed with specific flavour profiles that evoke positive emotions or offer a more intense and satisfying sensory experience.

### 4.3. Limitations and Future Research

The findings of this research offer valuable insights into the preferences of raw cashew nut consumers; however, there are several limitations to consider. Firstly, the sample size (n = 80) and the specific participant group (students and staff of the Universitat Politècnica de València) may restrict the generalisability of the results to broader populations. Secondly, the study’s focus on only two private-label brands limits the complexity of the statistical analyses that can be conducted, such as multivariate comparisons.

Future research could benefit from exploring additional factors that influence consumer preferences and emotional responses, such as geographical origin, processing methods, packaging, and willingness to pay. Furthermore, increasing the sample size and diversifying the participant profiles could provide a more comprehensive understanding of consumer preferences.

## 5. Conclusions

The combination of implicit consumer neuroscience methodologies with explicit sensory analysis techniques has provided a holistic view of consumer preferences toward raw cashew nuts. The convergence of results between objective measurements of brain activity (FAA, TE, and nSCR) and participants’ subjective evaluations reinforces the validity of the findings and underscores the importance of considering both conscious and unconscious responses in food perception research. Notably, this study represents one of the first applications of EEG and EDA to compare private-label nut brands, addressing a critical gap in the literature where nuts have been underrepresented in neuroscientific consumer studies.

From a practical perspective, these results demonstrate the potential of consumer neuroscience to inform targeted strategies for product optimisation and marketing. Additionally, the integration of penalty analysis with biometric metrics provides a framework for identifying sensory attributes that require prioritisation in product development.

The authors of this study believe that the results of this research demonstrate the potential of consumer neuroscience to provide valuable information for the optimisation and development of new food products and the improvement of marketing and communication strategies in the food industry.

## Figures and Tables

**Table 1:** Implicit measures mean to assess consumers’ valence (FAA) and arousal level (nSCR) for the two private labels studied.

Attributes	FAA (EEG)			nSCR (EDA)		
	PLA	PLB	*p*-Value	PLA	PLB	*p*-Value
Appearance	−0.238	−0.251	0.869	6.038	6.188	0.519
Aroma	−0.204	−0.217	0.106	7.914	8.139	<0.0001 *
Flavour	−0.247	−0.241	<0.0001 ***	4.576	4.388	0.189

Wilcoxon test significant differences * for *p* < 0.05, *** *p* < 0.0001. FAA: Higher values (closer to zero for negatives) indicate stronger approach motivation. nSCR: Higher values indicate increased physiological arousal.

**Table 2:** Task engagement (dB) mean of consumer interest during the evaluation of the appearance, aroma, and flavour of both private labels.

Attributes	Task Engagement (dB)		
	PLA	PLB	*p*-Value
Appearance	5.366	5.174	0.729
Aroma	8.793	8.358	<0.0001 ***
Flavour	8.530	9.099	0.037 *

Wilcoxon test significant differences for * *p* < 0.05, *** *p* < 0.0001. TE: Higher values indicate greater cognitive workload and task engagement.

**Table 3:** Results of the hedonic scores (1–9 points) mean of raw cashew nut attributes evaluated by consumers for each private label.

Attributes	PLA	PLB	*p*-Value
Appearance	6.088	6.488	0.120
Aroma	5.544	6.329	0.001 **
Flavour	6.325	6.838	0.060
Texture	6.638	6.988	0.065
Overall liking	6.363	6.950	0.017 *

Wilcoxon test significant differences for * *p* < 0.05, ** for *p* < 0.01.

**Table 4:** Percentage of consumers (n = 80) who overall chose each sample of raw cashew nuts.

	Paired Test	
	PLA	PLB
**%**	28 (35.00%)	52 (65.00%)

Minimum number of consumers = 50 for n = 80 and α = 0.05 according to ISO 5495:2005 Appendix A Table A2.

## Data Availability

The original contributions presented in the study are included in the article. Should further inquiries necessitate additional information, the corresponding author can be contacted directly.

## Abbreviations

The following abbreviations are used in this manuscript:

EDA	Electrodermal activity
EEG	Electroencephalography
FAA	Frontal alpha asymmetry
nSCR	Number of skin conductance response
JAR	Just about right
PLA	Private label A
PLB	Private label B
TE	Task engagement

## Appendix A

**Table A1:** Results of multiple comparison of penalty analysis from PLA sample.

Attributes	Level	Frequencies	%	Sum (Value)	Average	Mean Drops	Standardised Difference	*p*-Value
	Too Small	10	12.50%	58.000	5.800	0.583		
SIZE	Just About Right	47	58.75%	300.000	6.383			
	Too Big	23	28.75%	151.000	6.565	−0.182	−0.509	0.613
	Too clear	18	22.50%	104.000	5.778	0.924	2.296	0.025
COLOUR	Just About Right	47	58.75%	315.000	6.702			
	Too dark	15	18.75%	90.000	6.000	0.702		
	Too weak	61	76.25%	375.000	6.148	1.281	3.154	0.002
AROMA	Just About Right	14	17.50%	104.000	7.429			
	Too intense	5	6.25%	30.000	6.000	1.429		
	Low intensity	43	53.75%	250.000	5.814	1.553	5.163	<0.0001
FLAVOUR	Just About Right	30	37.50%	221.000	7.367			
	Too intense	7	8.75%	38.000	5.429	1.938		
	Too soft	25	31.25%	144.000	5.760	1.069	3.152	0.002
TEXTURE	Just About Right	41	51.25%	280.000	6.829			
	Too hard	14	17.50%	85.000	6.071	0.758		

**Table A2:** Results of multiple comparison of penalty analysis from PLB sample.

Variable	Level	Frequencies	%	Sum (Value)	Average	Mean Drops	Standardised Difference	*p*-Value
	Too Small	4	5.00%	25.000	6.250	0.903		
SIZE	Just About Right	59	73.75%	422.000	7.153			
	Too Big	17	21.25%	109.000	6.412	0.741	2.098	0.039
	Too clear	36	45.00%	249.000	6.917			
COLOUR	Just About Right	36	45.00%	249.000	6.917			
	Too dark	8	10.00%	58.000	7.250	−0.333		
	Too weak	36	45.00%	255.000	7.083	−0.240	−0.656	0.514
AROMA	Just About Right	32	40.00%	219.000	6.844			
	Too intense	12	15.00%	82.000	6.833	0.010		
	Low intensity	26	32.50%	162.000	6.231	1.325	4.153	<0.0001
FLAVOUR	As I like it	45	56.25%	340.000	7.556			
	Too intense	9	11.25%	54.000	6.000	1.556		
	Too soft	18	22.50%	111.000	6.167	1.199	3.265	0.002
TEXTURE	Just About Right	52	65.00%	383.000	7.365			
	Too hard	10	12.50%	62.000	6.200	1.165		
